# Nanoparticulate Tubular Immunostimulating Complexes: Novel Formulation of Effective Adjuvants and Antigen Delivery Systems

**DOI:** 10.1155/2017/4389525

**Published:** 2017-07-20

**Authors:** Nina Sanina, Natalia Chopenko, Andrey Mazeika, Eduard Kostetsky

**Affiliations:** Department of Biochemistry, Microbiology and Biotechnology, Far Eastern Federal University, Sukhanova St. 8, Vladivostok 690091, Russia

## Abstract

New generation vaccines, based on isolated antigens, are safer than traditional ones, comprising the whole pathogen. However, major part of purified antigens has weak immunogenicity. Therefore, elaboration of new adjuvants, more effective and safe, is an urgent problem of vaccinology. Tubular immunostimulating complexes (TI-complexes) are a new type of nanoparticulate antigen delivery systems with adjuvant activity. TI-complexes consist of cholesterol and compounds isolated from marine hydrobionts: cucumarioside A2-2 (CDA) from* Cucumaria japonica* and monogalactosyldiacylglycerol (MGDG) from marine algae or seagrass. These components were selected due to immunomodulatory and other biological activities. Glycolipid MGDG from marine macrophytes comprises a high level of polyunsaturated fatty acids (PUFAs), which demonstrate immunomodulatory properties. CDA is a well-characterized individual compound capable of forming stable complex with cholesterol. Such complexes do not possess hemolytic activity. Ultralow doses of cucumariosides stimulate cell as well as humoral immunity. Therefore, TI-complexes comprising biologically active components turned out to be more effective than the strongest adjuvants: immunostimulating complexes (ISCOMs) and complete Freund's adjuvant. In the present review, we discuss results published in series of our articles on elaboration, qualitative and quantitative composition, ultrastructure, and immunostimulating activity of TI-complexes. The review allows immersion in the history of creating TI-complexes.

## 1. Introduction

The appearance of idea about subunit vaccines seems to be the next important step in the history of vaccinology following the first experiment of active immunization in Europe, which was successfully carried out by E. Jenner in 1796. The application of subunit antigens in the prophylactic vaccination is the most effective way to enhance safety and protectiveness of modern vaccines. Subunit vaccines comprise one or few microbial antigens determining the occurrence of protective immune response of macroorganism, whereas the traditional vaccines comprise killed or attenuated pathogens. Therefore, the use of subunit vaccines allows eliminating many undesirable side effects associated with insufficient inactivation of the pathogen, the ability of attenuated pathogens to reverse to a wild virulent strain or to induce the related diseases of people with immunodeficiency or weakened health. Inactivation of pathogens may result in the destruction of epitopes that are important for a protective response [1]. Moreover, microorganisms may content antigens, which are able to induce such undesirable response as production of blocking antibodies preventing binding of functional bactericidal antibodies to the microbial epitopes.

However, individual pure antigens such as the surface proteins of viruses and bacteria usually reveal weak immune response that is insufficient to protect macroorganisms [2]. To enhance immunogenicity of subunit antigens, significant efforts have been made to elaborate more effective and safe new adjuvants and the respective ways of the antigen presentation to immunocompetent cells. Ideally, adjuvant should be safe and effective [3, 4]. Therefore, only few of them are suitable for medical and veterinary vaccines in spite of a wide arsenal of available adjuvants.

The present review summarizes literature data on properties of TI-complexes. These complexes consisting of lipid, cholesterol, and saponin [5] like their prototype, matrix of immunostimulating complex (ISCOMATRIX®) [3], are the most promising nanoparticulate adjuvant systems for the creation of effective and safe vaccines. In the present review, we discuss results published in series of our articles on elaboration, qualitative and quantitative composition, ultrastructure, and immunostimulating activity of TI-complexes. The review allows immersion in the history of creating TI-complexes. Elaborated novel formulation of adjuvant and antigen carrier allows using TI-complex as a universal adjuvant and antigen carrier for the creation of effective and safe anti-infectious subunit vaccines.

## 2. ISCOMATRIX as a Prototype of Tubular Immunostimulating Complex

Nanoparticulate ISCOMATRIX, which plays the role of both adjuvant and antigen carrier, is the most promising adjuvant-delivery system. The advantages of these adjuvant nanoparticles arise due to presentation of antigens as a part of the multidimensional form simulating microorganism. The high-performance nanoparticles of ISCOMATRIX induce both high levels of antibodies and the balanced Th1/Th2 response [4] that is important for neutralization of intracellular pathogens. ISCOMATRIX unlike more known ISCOMs [6] does not comprise antigen [7]. Therefore, the application field of ISCOMATRIX is essentially wider compared with ISCOMs, because it is not limited to the use of amphiphilic or hydrophobic membrane proteins only. ISCOMATRIX and the typical ISCOM usually comprise phospholipids from the egg yolk as well as cholesterol and Quil A, which is a heterogeneous mixture of more than 100 saponins extracted from the bark of* Quillaja saponaria*. Quil A possesses adjuvant activity [8]. They form virus-like spherical nanoparticles of 40–100 nm in diameter [9]. High immunostimulating activity of ISCOMATRIX is obviously related to the unique nanoparticulate structure of this adjuvant.

Quil A is suitable for veterinary applications, but not for human application [10]. The adjuvant activity of Quil A connected with the ability of these saponins to form complexes with membrane cholesterol, inducing inflammation at the injection site. In turn, this leads to the migration of lymphocytes and macrophages to the focus of inflammation that provides more effective presentation of antigen and the activation of immunocompetent cells [4]. However, Quil A is responsible for the by-effects connected with the lytic properties, painful effects, and molecular instability leading to degradation at physiological pH [11]. The complexation of Quil A with cholesterol in ISCOMATRIX was shown to remove hemolytic activity of saponin [4]. Despite significantly higher efficacy of ISCOMATRIX, compared with liposomes and attenuated virus vaccines, further studies aimed at creating novel formulation of adjuvant antigen delivery systems continue.

## 3. Elaboration of Tubular Immunostimulating Complex

ISCOMATRIX is a flexible structure, which allows replacing their main components on structure-related compounds [5]. Therefore principally new biologically active, well-characterized components, glycoglycerolipids and saponins from marine hydrobionts, were proposed to modify and optimize ISCOMATRIX vehicles for microbial antigens [12].

### 3.1. Modifications of ISCOMATRIX by Glycoglycerolipids from Marine Macrophytes

**Figure 1:**
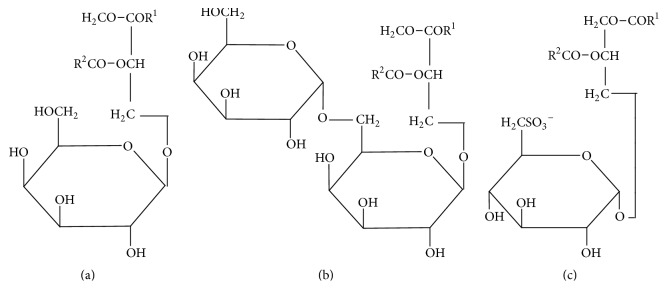
Chemical structures of glycoglycerolipids MGDG (a), DGDG (b), and SQDG (c). R1 and R2: hydrocarbon chains of fatty acid residues ((–CH_2_)_n_–CH_3_).

As a source of the lipid constituents, marine macrophytes (macroalgae and seagrasses) have many benefits. This widespread and phylogenetically diverse group of plants attracts the attention of nutritionists, pharmacists, and biotechnologists in particular due to a high content of glycoglycerolipids MGDG, digalactosyldiacylglycerol (DGDG), and sulfoquinovosyldiacylglycerol (SQDG) (Figure 1). Glycoglycerolipids of marine macrophytes possess different biological activities [13]: antioxidant [14], antivirus [15, 16], and anticancer [17, 18], and other types of biological activity [19]. MGDG reveals adjuvant activity, which is higher than the effect of Freund's complete adjuvant [20].

Moreover, glycolipids of marine macrophytes contain a high level of PUFAs which are precursors of various oxylipins [21] and, therefore, may exhibit biological activity [22, 23]. In particular, high content of PUFAs was found in MGDG of marine macrophytes [13]. PUFAs can reveal immunomodulatory properties in vitro and in vivo, affecting production of cytokines, proliferation of lymphocytes, phagocytosis, apoptosis, and so forth. The effect of PUFAs on the innate and adaptive immunity may be related to different molecular mechanisms. While the main functions of PUFAs are connected with their participation in metabolism of eicosanoids, expression of genes, signaling, and the membrane organization. The best comprehension was reached about the effect of PUFAs on production of eicosanoids. So, n-6 PUFAs are precursors of eicosanoids possessing properties of important regulators of the cell functions with inflammatory effects. In contrast, typical n-3 PUFAs, docosahexaenoic and eicosapentaenoic acids, show anti-inflammatory properties [24]. PUFAs exert immunomodulatory effects [25], in particular, by interfering in T-cell activation. n-3 PUFAs can convert proinflammatory Th1 phenotype into Th2 phenotype which is considered as an anti-inflammatory one.

**Figure 2:**
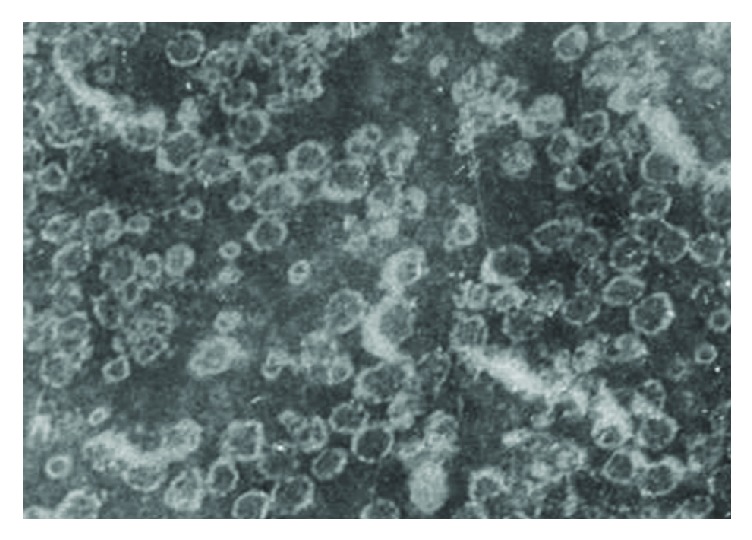
Electron micrograph of nanoparticles obtained after modification of ISCOMATRIX by MGDG from* Laminaria japonica* (magnification ×189000), taken with permission from [20].

At the first stage of the ISCOMATRIX modification, glycoglycerolipids from macroalgae* Laminaria japonica* were used to substitute immunologically inert phospholipids. Among three glycoglycerolipids (MGDG, DGDG, and SQDG), only MGDG was able to form ISCOM-like superstructures unlike DGDG and SQDG [20]. Particles of the resulting complexes had a classical vesicular morphology (Figure 2), which was typical for ISCOMATRIX, though they were 2 times less than phosphatidylcholine-containing ISCOMATRIX.

It is necessary to note that MGDG from marine macrophytes, which is enriched in PUFAs, is able to self-organize in hexagonal inverted superstructure, HII. In turn, SQDG and DGDG form lamellar and isotropic phase, respectively [26]. Hence, the superstructures, as well as comparatively small hydrophilic-lipophilic balance, are essential for the ability of lipids to form ISCOM-like particles [20]. It is also important that the phase transition temperature of MGDG from* L. japonica* was remarkably lower compared with ones of DGDG and SQDG. In turn, this correlated with unsaturation indexes of the studied glycolipids [26]. In the biological systems, the substitution of phospholipids for foreign glycolipids [27] is even accompanied by the improvement of some functional properties both of membrane-associated proteins and cells as a whole.

The study of immunostimulating activity showed the low adjuvant activity of phospholipid (phosphatidylcholine) ISCOM and MGDG-ISCOM for OmpF-like porin from enteropathogen* Yersinia pseudotuberculosis* (YOmpF) [20].

### 3.2. Modifications of ISCOMATRIX by Holothurian Triterpene Glycosides

Due to the main adjuvant function of Quil A in ISCOMATRIX, the next stage of modification consisted in the substitution of Quil A for triterpene glycoside from holothurian to enhance adjuvant activity of immunostimulating complexes. These saponins are characterized by the high adjuvant [28], being antitumor [29], and other [30] biological activities.

**Figure 3:**
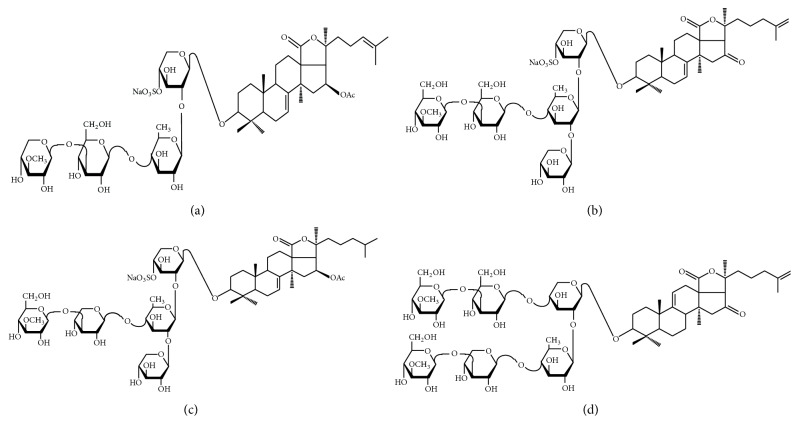
Structural formulas of triterpene glycosides extracted from holothurians: (a) cucumarioside G_1_, (b) CDA, (c) frondoside A, and (d) holotoxin A_1_.

Triterpene glycosides belong to a large group of compounds of glycoside nature with historically established name “saponins” (from the Latin “sapo,” soap) [31]. Molecule of saponin consists of the carbohydrate part and the aglycone, which is termed sapogenin. According to aglycone structure, they are divided into two groups: steroidal and triterpenoid saponins. Triterpenoid saponins are widely spread in plants in contrast to steroidal glycosides. Among the animals, triterpenoid saponins are broadly distributed in echinoderms [32] and are found in some sponges [33]. By the number of monosaccharide units, they are divided into monosides, biosides, triosides, tetraosides, pentaosides, and oligosides. Some saponins are bisdesmosides, that is, have two saccharide chains. Saccharide chains are linear or branched.* C. japonica* contents are very complex mixture of triterpene glycosides with closely related structure, which differs by the structure of carbohydrate chains and by the number and position of sulfate groups. Substances of one group differ in the structure of aglycones [34]. The common features for all isolated cucumariosides are the presence of pentasaccharide branched at the second monosaccharide unit (quinovose), by the sulfate group at position 4 of xylose residue, and at 7(8)-double bond in the aglycone [32] (Figure 3). CDA is the major monosulfated triterpene glycoside (Figure 3(b)).

#### 3.2.1. Biological Activity of Holothurian Triterpene Glycosides

Superlow doses of CDA stimulate both the cell and humoral immune responses. Triterpene glycosides of holothurians are able to stimulate effectively lysosomal activity of peritoneal macrophages of mice. Moreover, monosulfated triterpene glycosides from* C. japonica* do not reveal mutagenic activity and are approved for practical use in veterinary. However, their use in the content of vaccines is not studied till now. Triterpene glycosides of holothurians have broad-spectrum biological activities. The mechanism of their action is connected with high affinity to cholesterol of membranes [28]. Glycosides of holothurians are capable of binding selectively with membrane sterols that result in the formation of ion channels [35]. These channels may serve as a signal to the launch and stimulation of various cellular processes [36].

Pharmacological effect of triterpene glycosides is connected with the aglycone whereas glycones are responsible for solubility of the molecules in water and influence the absorbability and penetration through the membranes [37]. Some of monosulfated triterpene glycosides of holothurian* C. japonica* have a pronounced ability to increase the natural resistance of the animals to bacterial infections [38]. Probably, the basis of this effect is the activation of the mononuclear phagocyte system, including enhanced phagocytic activity of macrophages, as cucumariosides themselves do not show antibacterial activity [39]. Glycosides of* C. japonica* enhance the interaction between T- and B-lymphocytes and humoral response in animals [38], favorably affect the proliferation of stem cells, and contribute to the antiviral defense.

CDA has a significant adjuvant effect, causing an increase in antibody production in response to corpuscular antigens and enhances the protective effect of some antibacterial vaccines [38].

#### 3.2.2. Complex of Triterpene Glycosides and Cholesterol as the Base of Tubular Immunostimulating Complexes

Similar to Quil A, triterpene glycosides of holothurians are able to form glycoside-sterol complexes with the high affinity to cholesterol [37]. As a result, glycosides become less toxic than their free forms [40]. The ability of some saponin to form intermolecular complexes with cholesterol is a prerequisite for the formation of supramolecular lipid-saponin complexes [8] based on the respective saponin.

Four triterpene glycosides (Figure 3) extracted from the following species of holothurians:* Apostichopus japonicus* (holotoxin A1 (HTA)),* C. frondoza* (frondoside A (FRA)),* Eupentacta fraudatrix* (cucumarioside G1 (CDG)),* C. japonica* (CDA), and preparation of total triterpene glycosides (CD) from* C. japonica* were studied to clarify their capacity to form supramolecular complexes with cholesterol in phosphate buffered saline (PBS). Properties of these triterpene glycosides were described earlier [41–43].

**Figure 4:**
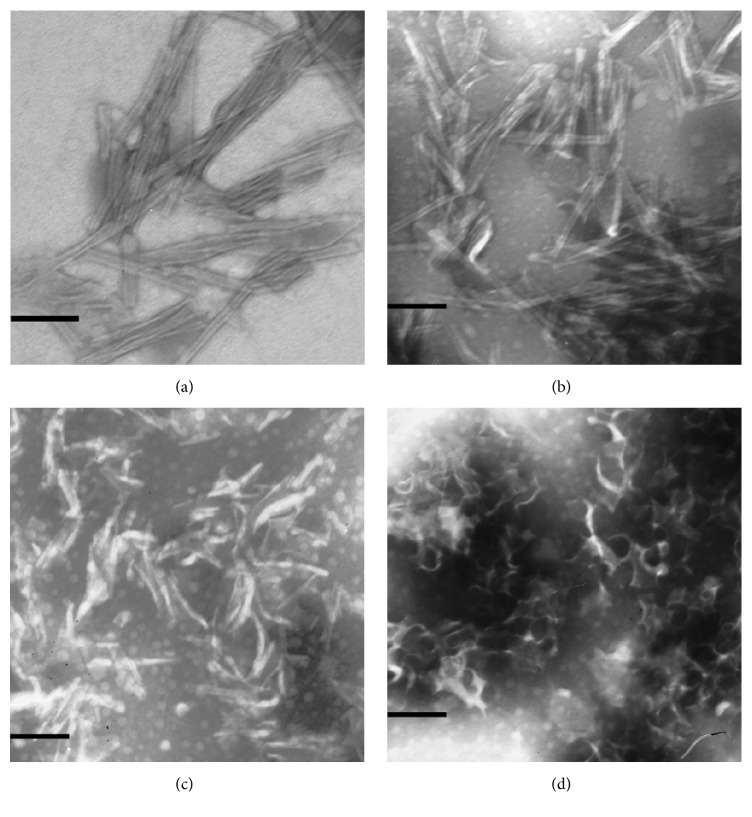
Electron micrographs of supramolecular complexes of cholesterol with CDA (a), HTA (b), FRA (c), and CDG (d). Bar is 100 nm, taken with permission from [44].

Complexes of cholesterol with triterpene glycosides extracted from holothurian were prepared by ultrasound dispersion of the cholesterol film in solution of triterpene glycosides [5]. The resulting complexes were examined by transmission electron microscopy using negative staining with a solution of phosphotungstic acid. All studied glycosides were able to form supramolecular complexes with cholesterol (Figure 4). However, the morphology of the particles of cholesterol-saponin complexes proved to be different in principle. For example, particles of CDA-cholesterol complexes, had a clear-cut tubular structure and a hydrophilic channel in the center. The formation of particles essentially depends on the ratio CDA-cholesterol. The increase of molar portion of cholesterol resulted in more pronounced tubular structure. Unlike CDA, CD forms complexes only at high molar portion of cholesterol.

The capacity of CDA and CD to form supramolecular structures was similar at molar ratio between glycoside and cholesterol 1 : 6. However, CD formed tubules with more outer and inner diameters that probably due to CD is the mixture of CDA and cucumarioside A4-2, whose amounts were approximately equal, as well as 10–20% of other monosulfated glycosides.

HTA and FRA also formed tubular supramolecular complexes with cholesterol. However, the expression degree of tubular structure decreased in the line CDA-HTA-FRA. CDG formed tubular supramolecular complexes without clearly expressed structure [44]. Then, CDA and HTA were chosen for the further development of antigen delivery system.

Earlier, Keukens et al. [45] described tubular supramolecular complexes of glycoalkaloids of plants from the family Solanaceae with membrane lipids. Similar to triterpene glycosides of holothurians, these compounds binding with steins, including cholesterol of biological membranes, exhibit strong hemolytic activity. Glycoalkaloids formed particles, which looked like long tubules of 20 nm in diameter. These particles are very similar to particles of complexes CDA-cholesterol and HTA with cholesterol that suggests the similarity of the molecular organization of complexes. Authors also suggested molecular model of complexes with these glycoalkaloids. They showed that the base of discovered particles is the monomeric glycoside-cholesterol complexes formed by the interaction of aglycone of glycoalkaloid with cholesterol [46]. Monomeric complexes have the conical form, of which narrow part is represented by a complex of aglycone of glycoalkaloid with cholesterol, while a wide part is formed of a carbohydrate chain of glycoside. The volume and the structure of carbohydrate chain of glycoside determine the width of the cone base. Monomeric complexes that interact with each other form a monolayer. Due to the conical shape of monomeric glycoside-cholesterol complexes, monolayer is spontaneously curved and closed in a tubule.

The reason of different morphology of particles formed by complexes of cholesterol with triterpene glycosides like with glycoalkaloids probably is the difference in the carbohydrate chains of triterpene glycosides [44]. Presumably, the monomeric complexes of cholesterol with glycoside like with glycoalkaloids have a conical shape, and their packing in the monolayer causes the formation of tubules. The analysis of the structural formulas of the studied triterpene glycosides (Figure 3) showed that the volumes of carbohydrate chains of CDA, HTA, and FRA are a lot more than ones of CDG due to branching and more carbohydrate residues. Probably, complexes of CDG with cholesterol unlike complexes of CDA, HTA, and FRA are narrow cones and therefore cannot provide the circuiting of their monolayer in tubules. The presence of sulfate residue at xylose does not seem critical for forming particles with a tubular structure. Aglycones of CDA and HTA unlike FRA and CDG do not contain acetyl residue at C-16 of the holostan system. This acetyl, probably, impedes the formation of uniform particles with a clearly defined structure upon complexation of FRA and CDG with cholesterol. Furthermore, CDA and HTA have a 25(26)-double bond in the aglycone and CDG has 24(25)-double bond, whereas FRA does not have a double bond at this location. The presence of this bond seems to determine the dependence of morphology of complexes formed by CDA, HTA, and CDG with cholesterol on the molar proportion of the latter in the system. Correspondingly, the absence of a double bond in this site of molecule provides the absence of such dependence for FRA.

**Figure 5:**
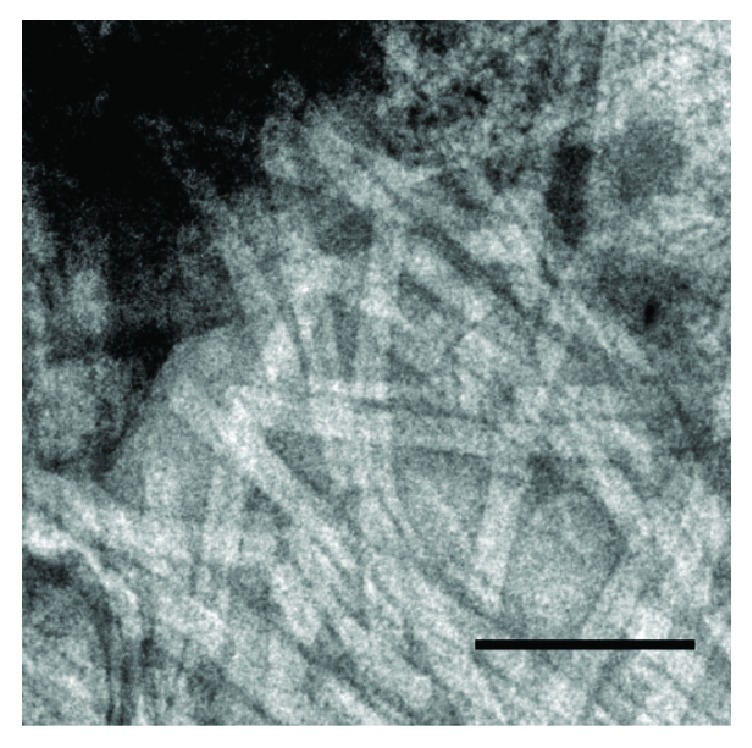
Electron micrograph of TI-complexes with weight ratio of CDA, cholesterol, and MGDG 6 : 2 : 4. Bar is 100 nm, taken with permission from [5].

The substitution not only of phospholipid component but also of saponin constituent element of ISCOMATRIX for CDA resulted in the formation of TI-complex, a new delivery system for subunit antigens. TI-complex is fundamentally different from the prototype both in composition and in morphology. Moreover, the use of MGDG isolated from different species of marine macrophytes had no effect on the formation of TI-complex. Initially, complex consisting of CDA, cholesterol, and MGDG from marine macrophytes with ratio between these components of 3 : 2 : 6 (by weight) was proposed, respectively [22]. TI-complexes were tubular particles with outer and inner diameters of about 13 nm and 4 nm, respectively, and the length of about 300 nm [42]. A new method for producing TI-complex [46], based on the method of hydration of lipid films [47], allowed obtaining homogeneous tubular particles, which is important for the reproducibility of the results of immunization. The weight ratio between CDA, MGDG, and cholesterol of 6 : 2 : 4 was found to be optimal for obtaining homogeneous TI-complexes. Then, this ratio of constituents allowed preventing the possibility of formation of particles other than the TI-complexes (Figure 5).

**Figure 6:**
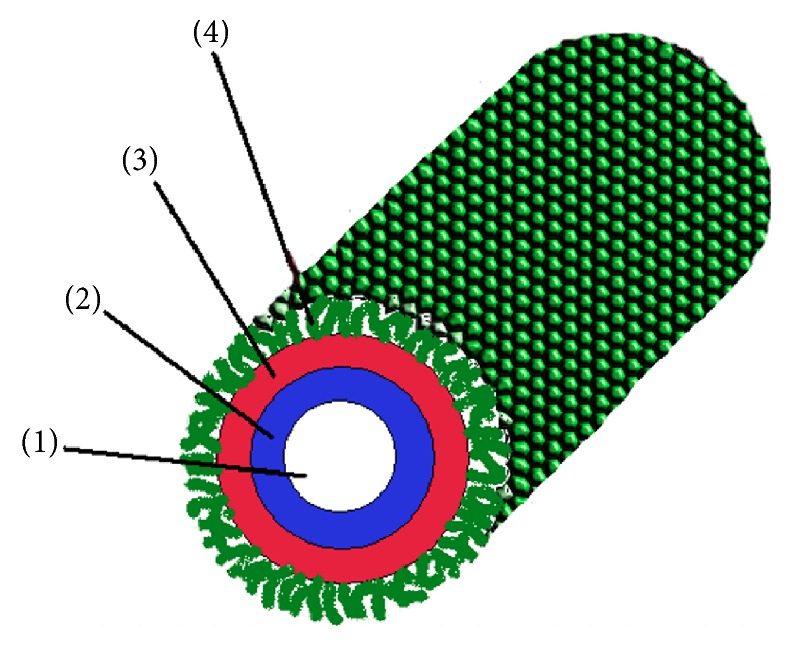
The model of the structure for tubular immunostimulating complex (TI-complex). (1) The inner channel, (2) the layer of MGDG from marine macrophytes, (3) the layer of cholesterol-aglycone complexes, and (4) the layer of carbohydrate chains of triterpene glycoside CDA, taken with permission from [46].

The positive staining of the particles by acidic hydrosol of iron, which electrostatically interacts with sulfate groups of carbohydrate chains, allows visualizing glycoside in the form of electron-dense substance in electron micrographs. Particles of TI-complexes were stained by hydrosol of iron outside iron, while the inner channel was not practically stained. This allows suggesting that carbohydrate chains of molecules of cucumariosides are exhibited chiefly on the outside of tubular particles. Such arrangement of the saponin molecules is consistent with the model of structure for the supramolecular complexes of glycoalkaloids from plants of the family Solanaceae [46] and allows suggesting that this model generally is proper for TI-complexes also (Figure 6).

At the example of YOmpF, it was shown that protein antigens could be effectively embedded in the given complex [48]. The structure of the antigen-containing complex did not differ from the original structure of TI-complex [49]. Experimental immunization of mice with YOmpF in the content of the TI-complexes resulted in a strong humoral immune response to the antigen. The immunogenicity of YOmpF incorporated in TI-complexes was significantly higher than the immunogenicity of this protein in the content of ISCOMs or in the mixture with Freund's complete adjuvant [5]. Porin in the content of TI-complexes induced 4 times higher level of anti-porin antibodies than individual antigen. TI-complexes do not possess hemolytic activity [50].

## 4. Effect of Components of TI-Complexes on Immunogenicity of Protein Antigens

As is known, polar lipid serves as a matrix for the protein antigen into immunostimulating complexes [3], whereas their saponin part is attributed to exclusively structural role. However, saponins, particularly triterpene glycosides, form molecular complexes with proteins [51]. The effects of polar lipids and triterpene glycosides isolated from various species of marine macrophytes and invertebrates on the immunogenicity and conformation of model protein antigens (porin from enteropathogen* Y. pseudotuberculosis *(YOmpF), uncleaved recombinant monomer of influenza virus H1N1 A/California/04/2009 (HA0), and human serum albumin (HSA)) were studied to understand the role of the chemical structure of these compounds in enhancing the effectiveness of vaccines based on the TI-complexes. The use of different proteins was dictated by the fact that the nature of this effect may depend on the properties of both interacting components [40].

In detail, results of these studies are described in respective publications [50, 51]. As a whole, it was shown that one of ways to achieve the desired immune response is a modulation of not so much fatty acid composition per se as microviscosity of MGDG. Moderate fluidity of MGDG which surrounds protein antigens probably is necessary for an optimal presentation of the antigenic determinants of the porin incorporated in TI-complexes. MGDGs with medium microviscosity gently stabilize protein conformation and provide maximal immunostimulating effect [52]. However, HA0 incorporated in TI-complexes induced a 2 times higher anti-HA0 antibodies level independently on physicochemical properties of MGDG, although the increase in the level of the cytokine GM-CSF by approximately 2 times corresponded to the protein conformational changes. In this case, the increase of MGDG's microviscosity resulted in conformational relaxation of hemagglutinin.

TI-complexes are able to enhance immunogenicity of the water-soluble protein antigen, HSA also. The content of anti-HSA antibodies increased approximately 2 times compared with effect of individual protein. This is coincides with data on the TI-complexes prototype, the ISCOMATRIX, which is able to play a role of an adjuvant for hydrophilic antigens nonincorporated in these nanoparticles. Observed effect simplifies the production of vaccines based on ISCOMATRIX and extends their application field. Phosphatidylcholine from the sea star* Distolasterias nipon*, which is characterized by the high ratio of n-3/n-6 PUFAs, is able to substitute MGDG in TI-complexes without disrupting their tubular morphology.

## 5. Conclusion

TI-complexes are a novel effective nanoparticulate adjuvant and antigen delivery system, which principally differ from their prototype, ISCOMATRIX, by their polar lipid and saponin constituents as well as their morphology. Adjuvant activity of TI-complexes is caused not only by immunostimulating properties of CDA but also by the chemical structure of polar lipid and their fluidity. Phospholipids from marine invertebrates can substitute MGDG from marine macrophytes in TI-complexes providing close level of their adjuvant activity. In this case, a polar lipid appears to be characterized by a high ratio of n-3/n-6 PUFAs due to the opposite effect of n-3 and n-6 PUFAs on the cells of immune system [24, 53]. TI-complexes are able to stimulate immune response both to the membrane and to water-soluble proteins. However, their adjuvant effect to the water-soluble proteins is likely lower compared with one to the membrane proteins, incorporated in TI-complexes. Moreover, each new antigen may need in special optimal lipid and saponin composition of TI-complexes to reveal maximal immunogenicity. Therefore, future studies should be aimed both at expanding spectrum of available membrane protein antigens, which are usually responsible for the protective activity, and at optimizing the composition of TI-complexes depending on the chosen antigen.

## Abbreviations

CD: Sum of monosulfated triterpene glycosides from* Cucumaria japonica*
CDA: Cucumarioside A_2_-2
CDG: Cucumarioside G
DGDG: Digalactosyldiacylglycerol
FRA: Frondoside A
GM-CSF: Granulocyte-macrophage colony-stimulating factor
HTA: Holotoxin A_1_
HSA: Human serum albumin
ISCOM: Immunostimulating complex
ISCOMATRIX: Matrix of immunostimulating complex
MGDG: Monogalactosyldiacylglycerol
PUFA: Polyunsaturated fatty acid
Quil A: Mixture of triterpene glycosides extracted from the bark of* Quillaja saponaria*
SQDG: Sulfoquinovosyldiacylglycerol
Th1: T helper cells of type 1
Th2: T helper cells of type 2
TI-complex: Tubular immunostimulating complex
HA0: Recombinant uncleaved hemagglutinin monomer
YOmpF: OmpF porin from* Yersinia pseudotuberculosis*.

## References

[1] Sanina N. M., Popov A. M. Prospects for the use of glycolipids and saponins for optimization of immunostimulatory complexes (ISCOM).

[2] Sanders M. T., Brown L. E., Deliyannis G., Pearse M. J. (2005). ISCOM™-based vaccines: the second decade. *Immunology and Cell Biology*.

[3] Sun H.-X., Xie Y., Ye Y.-P. (2009). ISCOMs and ISCOMATRIX™. *Vaccine*.

[4] Lövgren Bengtsson K., Morein B., Osterhaus A. D. (2011). ISCOM technology-based Matrix M™ adjuvant: success in future vaccines relies on formulation. *Expert Review of Vaccines*.

[5] Kostetsky E. Y., Sanina N. M., Mazeika A. N., Tsybulsky A. V., Vorobyeva N. S., Shnyrov V. L. (2011). Tubular immunostimulating complex based on cucumarioside A_2_-2 and monogalactosyldiacylglycerol from marine macrophytes. *Journal of Nanobiotechnology*.

[6] Morein B., Sundquist B., Höglund S., Dalsgaard K., Osterhaus A. (1984). Iscom, a novel structure for antigenic presentation of membrane proteins from enveloped viruses. *Nature*.

[7] Pearse M. J., Drane D. (2005). ISCOMATRIX® adjuvant for antigen delivery. *Advanced Drug Delivery Reviews*.

[8] Kersten G. F. A., Crommelin D. J. A. (1995). Liposomes and ISCOMS as vaccine formulations. *BBA - Reviews on Biomembranes*.

[9] Wikman M., Friedman M., Pinitkiatisakul S. (2006). Achieving directed immunostimulating complexes incorporation. *Expert Review of Vaccines*.

[10] Barr I. G., Sjölander A., Cox J. C. (1998). ISCOMs and other saponin based adjuvants. *Advanced Drug Delivery Reviews*.

[11] Pedebos C., Pol-Fachin L., Pons R., Teixeira C. V., Verli H. (2014). Atomic model and micelle dynamics of QS-21 saponin. *Molecules*.

[12] Popov A. M., Lee I. A., Kostetsky E. Y. Carrier of antigen.

[13] Sanina N. M., Goncharova S. N., Kostetsky E. Y. (2004). Fatty acid composition of individual polar lipid classes from marine macrophytes. *Phytochemistry*.

[14] Matsufuji M., Nagamatsu Y., Yoshimoto A. (2000). Protective effects of bacterial glyceroglycolipid M874B against cell death caused by exposure to heat and hydrogen peroxide. *Journal of Bioscience and Bioengineering*.

[15] Gustafson K. R., Cardellina J. H., Fuller R. W. (1989). AIDS-antiviral sulfolipids from cyanobacteria (Blue-Green Algae). *Journal of the National Cancer Institute*.

[16] Ohta K., Mizushina Y., Hirata N. (1998). Sulfoquinovosyldiacylglycerol, KM043, a new potent inhibitor of eukaryotic DNA polymerases and HIV-reverse transcriptase type 1 from a marine red alga, gigartina tenella. *Chemical and Pharmaceutical Bulletin*.

[17] Morimoto T., Nagatsu A., Murakami N. (1995). Anti-tumour-promoting glyceroglycolipids from the green alga, chlorella vulgaris. *Phytochemistry*.

[18] Eitsuka T., Nakagawa K., Igarashi M., Miyazawa T. (2004). Telomerase inhibition by sulfoquinovosyldiacylglycerol from edible purple laver *(Porphyra yezoensis)*. *Cancer Letters*.

[19] Wu J., Long L., Song Y. (2005). A new unsaturated glycoglycerolipid from a cultured marine dinoflagellate *Amphidinium carterae*. *Chemical and Pharmaceutical Bulletin*.

[20] Lee I. A., Popov A. M., Sanina N. M. (2004). Morphological and immunological characterization of immunostimulatory complexes based on glycoglycerolipids from *Laminaria japonica*. *Acta Biochimica Polonica*.

[21] Tilley S. L., Coffman T. M., Koller B. H. (2001). Mixed messages: modulation of inflammation and immune responses by prostaglandins and thromboxanes. *Journal of Clinical Investigation*.

[22] Calder P. C. (2009). Polyunsaturated fatty acids and inflammatory processes: new twists in an old tale. *Biochimie*.

[23] Calder P. C. (2015). Marine omega-3 fatty acids and inflammatory processes: effects, mechanisms and clinical relevance. *Biochimica et Biophysica Acta—Molecular and Cell Biology of Lipids*.

[24] Shaikh S. R., Edidin M. (2006). Polyunsaturated fatty acids, membrane organization, T cells, and antigen presentation. *American Journal of Clinical Nutrition*.

[25] Zhang P., Smith R., Chapkin R. S., McMurray D. N. (2005). Dietary (n-3) polyunsaturated fatty acids modulate murine Th1/Th2 balance toward the Th2 pole by suppression of Th1 development. *The Journal of Nutrition*.

[26] Sanina N. M., Goncharova S. N., Kostetsky E. Y. (2008). Seasonal changes of fatty acid composition and thermotropic behavior of polar lipids from marine macrophytes. *Phytochemistry*.

[27] Wikström M., Xie J., Bogdanov M. (2004). Monoglucosyldiacylglycerol, a foreign lipid, can substitute for phosphatidylethanolamine in essential membrane-associated functions in *Escherichia coli*. *Journal of Biological Chemistry*.

[28] Kalinin V. I., Aminin D. L., Avilov S. A., Silchenko A. S., Stonik V. A. (2008). Triterpene glycosides from sea cucucmbers (holothurioidea, echinodermata). Biological activities and functions. *Studies in Natural Products Chemistry*.

[29] Menchinskaya E. S., Pislyagin E. A., Kovalchyk S. N. (2014). Antitumor activity of cucumarioside A_2_-2. *Chemotherapy*.

[30] Aminin D. L., Zaporozhets T. S., Adryjashchenko P. V., Avilov S. A., Kalinin V. I., Stonik V. A. (2011). Radioprotective properties of cumaside, a complex of triterpene glycosides from the sea cucumber *Cucumaria japonica* and cholesterol. *Natural Product Communications*.

[31] Vincken J.-P., Heng L., de Groot A., Gruppen H. (2007). Saponins, classification and occurrence in the plant kingdom. *Phytochemistry*.

[32] Kalinin V. I., Silchenko A. S., Avilov S. A. (2016). Taxonomic significance and ecological role of triterpene glycosides from holothurians. *Biology Bulletin*.

[33] Colorado J., Muñoz D., Marquez D. (2013). Ulososides and urabosides—triterpenoid saponins from the caribbean marine sponge ectyoplasia ferox. *Molecules*.

[34] Silchenko A. S. (2005). *New triterpene glycosides from 8 specias of holothurians from families Holothuriidae, Stichopodidae, Synallactidae and Cucumariidae [Ph.D. thesis]*.

[35] Li X., Roginsky A. B., Ding X.-Z. (2008). Review of the apoptosis pathways in pancreatic cancer and the anti-apoptotic effects of the novel sea cucumber compound, frondoside A. *Annals of the New York Academy of Sciences*.

[36] Likhatskaya G. N. (2011). *Triterpene and sterioidal glycosides and membranes. Molecular mechanisms of interactions with membranes*.

[37] Popov A. M. (2003). *Biological activity and mechanism of action of secondary metabolites from terrestrial plants and marine invertebrates [Dr. Sci. Thesis]*.

[38] Avilov S. A. (2000). *Triterpene glycosides from holothurians of an order Dendrochirotida [Dr. Sci. Thesis]*.

[39] Aminin D. L., Pinegin B. V., Pichugina L. V. (2006). Immunomodulatory properties of Cumaside. *International Immunopharmacology*.

[40] Lee L. A., Popov A. M., Kostetsky E. Ya., Sanina N. M., Mazeyka A. N., Boguslavskii V. M. (2008). Membranotropic effect of some triterpene glycosides possessing immunostimulating properties. *Biophysics*.

[41] Kalinin V. I., Silchenko A. S., Avilov S. A., Stonik V. A., Smirnov A. V. (2005). Sea cucumbers triterpene glycosides, the recent progress in structural elucidation and chemotaxonomy. *Phytochemistry Reviews*.

[42] Aminin D. L., Koy C., Dmitrenok P. S. (2009). Immunomodulatory effects of holothurian triterpene glycosides on mammalian splenocytes determined by mass spectrometric proteome analysis. *Journal of Proteomics*.

[43] Aminin D. L., Menchinskaya E. S., Pisliagin E. A., Silchenko A. S., Avilov S. A., Kalinin V. I. (2015). Anticancer activity of sea cucumber triterpene glycosides. *Marine Drugs*.

[44] Mazeyka A. N., Popov A. M., Kalinin V. I., Avilov S. A., Silchenko A. S., Kostetsky E. Y. (2008). Complexation between triterpene glycosides of holothurians and cholesterol is the basis of lipid-saponin carriers of subunit protein antigens. *Biophysics*.

[45] Keukens E. A. J., de Vrije T., van den Boom C. (1995). Molecular basis of glycoalkaloid induced membrane disruption. *Biochimica et Biophysica Acta—Biomembranes*.

[46] Mazeyka A. N., Kostetsky E. Y., Sanina N. M., Popov A. M., Kalinin V. I., Li I. A. (2013). Elaboration of immune stimulating lipid-saponin subunit antigen carrier based on glycolipid monogalactosyldiacylglycerol from sea macrophytes and triterpene glycosides from *Cucumaria japonica*. *Biophysics (Russian Federation)*.

[47] Copland M. J., Rades T., Davies N. M. (2000). Hydration of lipid films with an aqueous solution of Quil A: a simple method for the preparation of immune-stimulating complexes. *International Journal of Pharmaceutics*.

[48] Kostetsky E. Y., Popov A. M., Sanina N. M., Lee I. A., Tsybulsky A. V., Shnyrov V. L. Carrier and adjuvant for antigens.

[49] Sanina N. M., Popov A. M., Lee I. A., Kostetsky E. Y., Tsybulsky A. V., Shnyrov V. L. Mode of forming of antigen carrier based on lipids from marine macrophytes and triterpene glycoside cucumarioside.

[50] Vorobyeva N. S., Mazeika A. N., Davydova L. A. (2015). The effects of triterpene glycosides and phospholipids from marine invertebrates in the composition of tubular immunostimulating complexes on the immunogenicity of human serum albumin. *Russian Journal of Marine Biology*.

[51] Sanina N. M., Vorobieva N. S., Novikova O. D. (2016). Lipid-induced changes in protein conformation as a means to regulate the immunogenicity of antigens incorporated in tubular immunostimulating complexes. *Biophysics (Russian Federation)*.

[52] Sanina N. M., Kostetsky E. Y., Shnyrov V. L. (2012). The influence of monogalactosyldiacylglycerols from different marine macrophytes on immunogenicity and conformation of protein antigen of tubular immunostimulating complex. *Biochimie*.

[53] Yaqoob P. (2003). Fatty acids as gatekeepers of immune cell regulation. *Trends in Immunology*.

